# Retraction: Arterial Stiffness and Obesity as Predictors of Diabetes: Longitudinal Cohort Study

**DOI:** 10.2196/108369

**Published:** 2026-08-19

**Authors:** JMIR Publications Editorial Office

**Affiliations:** 1 JMIR Publications Toronto, ON

The JMIR Publications Editorial Office is retracting the article “Arterial Stiffness and Obesity as Predictors of Diabetes: Longitudinal Cohort Study” [1] due to concerns regarding the integrity of the peer review process.

Author Lin Liu responded to the concerns, stating that there were undisclosed conflicts of interest with the reviewers but asserted these did not impact the peer review process for the article.

However, the Editorial Office and editor in chief have determined that there is sufficient evidence indicating that the peer review process for this article was compromised.

The authors did not respond to the decision to retract the article.
